# Poverty and Intellectual Development in Childhood.

**DOI:** 10.23889/ijpds.v9i5.2614

**Published:** 2024-09-18

**Authors:** Gordon Milligan, Erum Masood, Phil Quinlan, Sam Cox, Tom Giles, Armando Mendez Villalon, Christian Cole

**Affiliations:** 1 University of Dundee; 2 University of Nottingham

## Objectives and Approach

The effects of the timing and duration of economic deprivation and maternal mental health on intellectual development is researched using unique linkable databases following almost 90 thousand children born in the Canadian province of Manitoba.

This paper studies such questions as:

- How important are differences in measurement of poverty in assessing intellectual development?
- How do various differences change over childhood?

## Results

Concentrating on those born in 2000-2002 generated a total of 7,424 children having scores on all six measures of intellectual development. Such person-specific information controlled for many individual factors and extended from ages 5 through 17.

Major differences were found in the scores associated with exposure to the two kinds of poverty and to poor maternal mental health.

In summary, differences emerge by age 5, with administration of the Early Development Index. These differences are largely the same at age 8, even though another measure (the Grade 3 Competencies Index) is used.

## Conclusions

If household poverty is taken as a definition of poverty, poverty seems much more important than maternal mental health in affecting a child’s intellectual development. However, if neighborhood poverty is used to define poverty, maternal mental health and poverty have effects on childhood intellectual development which are more similar. Definition plays a critical role in interpretation.

## Implications

These population-based data, with multiple measures and multiple time points, suggest many analytical possibilities. If childhood conditions are included, the list of medical conditions which might correlate with intellectual development multiplies.

